# Insulin Resistance of Normal Weight Central Obese Adolescents in Korea Stratified by Waist to Height Ratio: Results from the Korea National Health and Nutrition Examination Surveys 2008–2010

**DOI:** 10.1155/2015/158758

**Published:** 2015-07-16

**Authors:** Won Kyoung Cho, Hyojin Kim, Hyun Young Lee, Kyung Do Han, Yeon Jin Jeon, In Ah Jung, Shin Hee Kim, Kyoung Soon Cho, So Hyun Park, Min Ho Jung, Byung-Kyu Suh

**Affiliations:** ^1^Department of Pediatrics, College of Medicine, The Catholic University of Korea, Seoul 137-701, Republic of Korea; ^2^Department of Biostatistics, College of Medicine, The Catholic University of Korea, Seoul 137-701, Republic of Korea

## Abstract

*Background*. To evaluate insulin resistance of normal weight central obese 13–18-year-old male and female adolescents stratified by waist to height ratio (WHR). *Methods*. Data were obtained from the Korea National Health and Nutrition Examination Survey (K-NHANES) conducted during 2008–2010. Central obesity was defined as that in the upper quartile of age and sex specific WHR. Subjects were classified into no central obesity normal weight (NW), central obesity normal weight (CONW), no central obesity overweight (OW), and central obesity overweight (COOW). *Results*. The prevalence of CONW was 9.6% (83/832) in female and 7.0% (61/909) in male. CONW showed higher levels of insulin (*P* < 0.006), HOMA-IR (*P* < 0.006), and ALT (*P* < 0.001) than NW in female. CONW had higher levels of insulin (*P* < 0.0001), HOMA-IR (*P* < 0.0001), and WBC count (*P* < 0.021) and lower level of HDL (*P* < 0.0001) than NW in male. WHR and BMI had similar significant correlations with MS components. CONW showed 2.5 times (95% confidence interval, 1.21–5.00) more likelihood to have high insulin resistance than NW in male. *Conclusions*. Screening for central obesity using WHR in clinical setting is recommended.

## 1. Introduction

The prevalence of childhood obesity has significantly increased worldwide and has become an important global public health issue [1]. Childhood obesity is a risk factor for cardiovascular disease and metabolic syndrome (MS) in adulthood and thus affects morbidity and mortality in later life [2]. Adipose tissue in the visceral fat has been considered a major contributing factor in insulin resistance as well as a risk of type 2 diabetes and coronary heart disease [3–5]. The World Health Organization (WHO) defines obesity as an excessive accumulation of fat to an extent that health may be impaired [6]. The most commonly used anthropometric tool to classify obesity is body mass index (BMI) [7]. However, BMI cannot distinguish between lean and fat mass [7, 8].

Recently, recognition of different subtypes of obesity including metabolically healthy but obese individuals and metabolically obese but normal weight individuals has been reported [9]. This suggests that individuals should not be classified as obese based solely on BMI. Indeed, a substantial proportion of BMI-defined normal weight showed cardiometabolic abnormalities that puts them at increased risk for future development of obesity-related diseases [8]. A previous study suggested that abdominal obesity may indicate higher cardiovascular disease risks even in normal weight women [10]. Therefore, screening for abdominal obesity in adolescents with normal weight is an important contribution to the prevention of obesity related disease.

Waist to height ratio (WHR) has been reported to be useful in screening for cardiovascular risk factor in children and adolescents [11–14]. This measure is easier to calculate than BMI, does not require tables, and can be used to diagnose visceral obesity, even in normal weight individuals [15]. WHR incorporates waist circumference as a measure of abdominal obesity and adjusts for an individual's size by dividing their height [16]. It combines the advantages of both BMI and waist to hip ratio by accounting for height and abdominal adiposity [17]. WHR further specifies cardiometabolic risk based on BMI percentile [18].

There are no reports of the characteristics or prevalence of subjects who are of normal weight by BMI but having central obesity when stratified by WHR in Korean adolescents. The aim of this study is to investigate the metabolic characteristics in Korean adolescents with central obesity but normal weight by BMI.

## 2. Methods

### 2.1. Data

Data were obtained from the Korea National Health and Nutrition Examination Surveys (K-NHANES) conducted from 2008 to 2010 by the Korean Ministry of Health and Welfare. K-NHANES is a cross-sectional survey based on stratified multistage probability samples of Korean households representing the civilian noninstitutionalized population. More details regarding study design and methods are provided elsewhere [19]. The K-NHANES data are publicly available; thus, this study did not require approval from the institutional review board.

### 2.2. Selection of Study Populations

Overall, 2280 subjects aged 13–18 years participated in the 2008–2010 K-NHANES. Among them, 411 participants were excluded due to incomplete information about anthropometric data (height and weight) and laboratory data. Of these 1869 subjects, 128 having below 5th percentile of BMI according to age and sex were excluded. Ultimately, our study population included 1741 adolescents (male = 909, female = 832).

### 2.3. Anthropometric and Laboratory Measurements

Height was measured using a stadiometer (SECA, Hamburg, Germany), and weight was measured with a balance beam scale (GL-6000-20, CASKOREA, Korea) while participants were wearing a standardized gown. Waist circumference (WC) was measured to the nearest 0.1 cm at the end of normal expiration, measuring from the narrowest point at the midline of the most lateral border of the right and left ileac crest [19]. Fasting plasma concentrations of glucose, insulin, triglyceride (TG), and high density lipoprotein (HDL), alanine aminotransferase (ALT), and aspartate aminotransferase (AST) were measured enzymatically using a Hitachi 747 chemistry analyzer (Daiichi, Tokyo, Japan) after subjects had participated in a minimum 8-hour overnight fast. White blood cell (WBC) count was obtained using XE-2100D (Sysmex, Kobe, Japan). Homeostasis model assessment-insulin resistance (HOMA-IR) was calculated as fasting glucose (in millimoles per liter) × fasting insulin (in milliunits per liter)/22.5, while blood pressure (BP) was calculated as the mean of three successive readings using a standard mercury sphygmomanometer (Baumanometer). Mean arterial pressure was also calculated.

### 2.4. Study Criteria

A subject was first classified as normal weight (5th–85th percentiles) or overweight (include obesity, ≥85th percentile) by BMI according to 2007 Korea Growth Charts [20]. There was no confirmed cut-off value of WHR for health risks of obesity in adolescents. So we defined the central obesity as those in the upper highest quartile (Q4) of age and sex specific WHR and the no central obesity as those below the highest quartile (Q1, Q2, and Q3) of WHR. Following these criteria, body composition groups were further classified into no central obesity normal weight (NW), central obesity normal weight (CONW), no central obesity overweight (OW), and central obesity overweight (COOW). We defined subjects as having high insulin resistance if they were in the upper highest quartile (Q4) according to age and sex of HOMA-IR measurements.

### 2.5. Statistical Analyses

All statistical analyses were performed using SAS Version 9.3 (SAS Institute Inc., Cary, NC). Sampling weights were incorporated to produce valid population estimates that accounted for the complex survey design of K-NHANES. Data are presented as mean ± standard error (SE) for continuous variables and frequency percentage for categorical variables. If necessary, logarithmic transformation was performed to achieve a normal distribution. The characteristics and laboratory data of body composition groups by sex were compared using ANOVA for continuous measures and chi-squared tests for categorical measures. Bonferroni corrections were used to assess the significance between 4 groups. Pearson's correlation coefficients between WHR or BMI and various parameters of the insulin resistance syndrome were calculated. Multivariate logistic regression models were used to compare the adjusted odds ratio (OR) and 95% confidence interval (CI) of having high insulin resistance among body composition groups after adjusting for potentially confounding variables. Multivariate analyses of the adjusted ORs of having high insulin resistance were first adjusted for age (model 1) and then age plus weight (model 2). Model 3 was adjusted for the variables in model 2 plus ALT. Results with *P* value less than 0.05 were considered significant.

## 3. Results

### 3.1. The Prevalence of NW, CONW, OW, COOW, and Overweight in 13–18-Year-Old Korean Adolescents

The prevalence of NW was 72.7% (604/832), CONW was 9.6% (83/832), OW was 2.5% (20/832), and COOW was 15.1% (125/832) in female adolescents. In male adolescents, the prevalence of NW was 72.3% (662/909), CONW was 7.0% (61/909), OW was 2.2% (21/909), and COOW was 18.5% (165/909). The prevalence of overweight (include obesity) was 20.7% in male and 17.6% in female in 13–18-year-old 1741 Korean adolescents (subjects below 5th percentile of BMI were excluded) (Tables 1 and 2). When subjects below 5th percentile of BMI were included, the prevalence of overweight (include obesity) was 17.6% in male and 15.1% in female in 13–18-year-old 2280 Korean adolescents using the data from 2008–2010 K-NHANES (data not shown).

### 3.2. Characteristic and Laboratory Data according to Four Body Composition Groups in 13–18-Year-Old Korean Adolescents

In female adolescents, CONW showed higher mean levels of weight (50.8 ± 0.2 versus 54.9 ± 1.0, *P* < 0.0001), BMI (19.7 ± 0.1 versus 21.7 ± 0.2, *P* < 0.0001), WC (65.8 ± 0.2 versus 75.1 ± 0.6, *P* < 0.0001), WHR (41.0 ± 0.1 versus 47.3 ± 0.2, *P* < 0.0001), insulin [11.4 (11–11.8) versus 12.9 (11.9–13.9), *P* < 0.006], HOMA-IR [2.5 (2.4–2.5) versus 2.8 (2.6–3.0), *P* < 0.006], and ALT [10.0 (9.7–10.3) versus 11.5 (10.8–12.3), *P* < 0.001] than NW. COOW showed higher mean levels of weight, BMI, WC, and WHR than OW (Table 1).

In male adolescents, CONW showed higher mean levels of weight (59.9 ± 0.4 versus 68.0 ± 1.2, *P* < 0.0001), BMI (20.3 ± 0.1 versus 23.7 ± 0.1, *P* < 0.0001), WC (70.1 ± 0.3 versus 82.2 ± 0.6, *P* < 0.0001), WHR (40.9 ± 0.1 versus 48.7 ± 0.3, *P* < 0.0001), insulin [10.6 (10.3–11.0) versus 13.6 (12.3–15.1), *P* < 0.0001], HOMA-IR [2.3 (2.3–2.4) versus 3.0 (2.7–3.3), *P* < 0.0001], ALT [12.8 (12.3–13.4) versus 17.0 (15.3–18.8), *P* < 0.0001], and WBC count [6.0 (5.9–6.1) versus 6.4 (6.1–6.8), *P* < 0.021] and lower mean level of HDL (51.8 ± 0.4 versus 47 ± 1.1, *P* < 0.0001) than NW. COOW had higher mean levels of BMI, WC, WHR, TG, and TG/HDL ratio and lower mean level of HDL than OW (Table 2). In both male and female adolescents, there were no significant differences in MS components between CONW and OW (data not shown).

### 3.3. Correlations between WHR or BMI and MS Components in 13–18-Year-Old Korean Adolescents

In female adolescents, WHR showed significant positive correlations with BMI (*r* = 0.84, *P* < 0.0001), SBP (*r* = 0.18, *P* < 0.0001), insulin (*r* = 0.30, *P* < 0.0001), HOMA-IR (*r* = 0.30, *P* < 0.0001), TG (*r* = 0.17, *P* < 0.0001), TG/HDL (*r* = 0.22, *P* < 0.0001), ALT (*r* = 0.32, *P* < 0.0001), and WBC (*r* = 0.16, *P* < 0.0001) and showed significant negative correlations with HDL (*r* = −0.22, *P* < 0.0001). BMI showed significant positive correlations with SBP (*r* = 0.18, *P* < 0.0001), insulin (*r* = 0.33, *P* < 0.0001), HOMA-IR (*r* = 0.32, *P* < 0.0001), TG (*r* = 0.16, *P* < 0.0001), TG/HDL (*r* = 0.22, *P* < 0.0001), ALT (*r* = 0.33, *P* < 0.0001), and WBC (*r* = 0.19, *P* < 0.0001) and showed significant negative correlations with HDL (*r* = −0.23, *P* < 0.0001).

In male adolescents, WHR showed significant positive correlations with BMI (*r* = 0.91, *P* < 0.0001), SBP (*r* = 0.27, *P* < 0.0001), insulin (*r* = 0.49, *P* < 0.0001), HOMA-IR (*r* = 0.47, *P* < 0.0001), TG (*r* = 0.32, *P* < 0.0001), TG/HDL (*r* = 0.35, *P* < 0.0001), ALT (*r* = 0.53, *P* < 0.0001), and WBC (*r* = 0.26, *P* < 0.0001) and showed significant negative correlations with HDL (*r* = −0.27, *P* < 0.0001). BMI showed significant positive correlations with SBP (*r* = 0.33, *P* < 0.0001), insulin (*r* = 0.48, *P* < 0.0001), HOMA-IR (*r* = 0.46, *P* < 0.0001), TG (*r* = 0.31, *P* < 0.0001), TG/HDL (*r* = 0.34, *P* < 0.0001), ALT (*r* = 0.52, *P* < 0.0001), AST (*r* = 0.19, *P* < 0.0001), and WBC (*r* = 0.27, *P* < 0.0001) and showed significant negative correlation with HDL (*r* = −0.27, *P* < 0.0001) (Table 3).

### 3.4. Correlations between WHR and MS Components of Normal and Overweight 13–18-Year-Old Korean Adolescents

In normal weight female adolescents, WHR exhibited a significant positive correlation with BMI (*r* = 0.67, *P* < 0.0001), insulin (*r* = 0.10, *P* < 0.014), HOMA-IR (*r* = 0.084, *P* < 0.025), TG/HDL ratio (*r* = 0.10, *P* < 0.015), and ALT (*r* = 0.23, *P* < 0.0001) and a significant negative correlation with HDL (*r* = −0.10, *P* < 0.017). In overweight female adolescents, WHR showed significant positive correlations with BMI (*r* = 0.75, *P* < 0.0001), SBP (*r* = 0.30, *P* < 0.001), insulin (*r* = 0.24, *P* < 0.0006), HOMA-IR (*r* = 0.26, *P* < 0.002), and ALT (*r* = 0.19, *P* < 0.034).

In normal weight male adolescents, WHR showed a significant positive correlation with BMI (*r* = 0.80, *P* < 0.0001), SBP (*r* = 0.10, *P* < 0.018), insulin (*r* = 0.27, *P* < 0.0001), HOMA-IR (*r* = 0.29, *P* < 0.0001), TG/HDL ratio (*r* = 0.14, *P* < 0.008), ALT (*r* = 0.32, *P* < 0.0001), and WBC (*r* = 0.15, *P* < 0.001) and a significant negative correlation with HDL (*r* = −0.17, *P* < 0.0001). In overweight male adolescents, WHR showed significant positive correlations with BMI (*r* = 0.84, *P* < 0.0001), insulin (*r* = 0.26, *P* < 0.001), HOMA-IR (*r* = 0.23, *P* < 0.003), TG (*r* = 0.23, *P* < 0.005), TG/HDL ratio (*r* = 0.23, *P* < 0.005), ALT (*r* = 0.33, *P* < 0.0001), AST (*r* = 0.27, *P* < 0.0007), and WBC (*r* = 0.17, *P* < 0.038) and a significant negative correlation with HDL (*r* = −0.17, *P* < 0.036) (Table 4).

### 3.5. Multivariate Logistic Regression Analyses of Having High Insulin Resistance among Body Composition Groups

In female adolescents, there was no significant difference in odds ratio of having upper highest quartile of age and sex specific HOMA-IR between NW and CONW or OW (model 3). Only COOW was 2.1 times (95% confidence interval [CI], 1.1–4.2) more likely to have high insulin resistance than NW after adjusting age, weight, and ALT (model 3).

In male adolescents, CONW was 2.5 times (95% CI, 1.2–5.0) more likely to have high insulin resistance than NW (model 3). However, there was no significant difference in odds ratio of having high insulin resistance between OW and NW. And COOW was 2.4 times (95% CI, 1.2–4.7) more likely to have high insulin resistance than NW after adjusting age, weight, and ALT (model 3) (Table 5).

## 4. Discussion

In the present study, we found that 9.6% of female and 7.0% of male are CONW which are classified as normal weight by BMI. In adult male and female of different ethnic groups, the cut-off value of WHR for health risks of obesity was 0.5 [14]. Since the height and waist circumference of children increases continually as they age, some reports suggest 0.5 could be a cut-off value of WHR for health risks of obesity in children [16, 21, 22]. However, there was no confirmed cut-off value of WHR for health risks of obesity in adolescents. The subjects in our study were aged 13–18 years and not homogenous in terms of the degree of maturation. Data from K-NHANES did not include information about sexual maturation rate. For adjusting heterogeneity in terms of the degree of maturation, we classified central obesity as that in the age and sex specific upper highest quartile (Q4) of WHR. Asians are known to have higher amounts of visceral adipose tissue than Caucasians [23]. Our results can be due to the impact of ethnicity. It is recommended to assess and compare the prevalence CONW using universal cut-off value of WHR for health risks of obesity in multiple ethnic adolescents in the near future.

In this study, the prevalence of overweight (include obesity) was 20.7% in male and 17.6% in female in 13–18-year-old 1741 Korean adolescents (those below 5th percentile of BMI were excluded). Our data on the prevalence of overweight (include obesity) were similar to the previous reports [24, 25]. Overall, about 30% of American adolescents and 25% of European adolescents were overweight or obese [26]. The prevalence of overweight in Korean adolescents was lower than America and Europe. However, the prevalence of obesity increased from 6.1% in 1997 to 11.3% in 2005 in boys and increased from 5.5% in 1997 to 8.0% in 2005 in girls [27].

We found that CONW showed poor MS components compared to NW and no significant difference in MS components compared to OW in both male and female adolescents. Metabolically obese but normal weight individuals were first described by Ruderman et al. [28]. A central obese but normal weight individuals who increased risks for MS has been reported [8, 29–31]. In Korean adults, high prevalence of cardiometabolic abnormalities among subjects with normal weight having central obesity was previously reported [32]. Our results suggest that more medical concerns for CONW classified normal weight by BMI are necessary even in adolescents.

We found that WHR and BMI had similar significant correlations with SBP, insulin, HOMA-IR, TG, TG/HDL, ALT, WBC, and HDL in Korean adolescents. WHR is recommended as an effective and precise anthropometric index to assess visceral adiposity and to predict insulin resistance [33]. Excess visceral fat is associated with high impaired suppression of free fatty acid (FFA) release and insulin resistance in muscle and liver [34, 35]. These results may suggest that WHR is comparable to BMI in predicting MS components in Korean adolescents.

In male, CONW was 2.5 times more likely to have high insulin resistance than NW after adjusting for age, weight, and ALT. This finding indicates that insulin resistance can be present even in adolescents classified as normal weight by BMI. However, the odds ratio having high insulin resistance of CONW than NW was not significant in female Korean adolescents. This result was inconsistent with previous report of other ethnic adolescents. Some report suggests that intra-abdominal obesity is associated with adverse cardiovascular risk factor in both male and female adolescents [36]. Other reports showed that central obesity index was only associated with insulin resistance in only female obese Caucasian adolescents [37]. Women have more subcutaneous fat, whereas men have more visceral fat which is already previously reported. However, there was no significant difference in visceral fat between female and male adolescents [38]. Visceral adipose tissue carries a greater risk for cardiovascular disorders than does subcutaneous adipose tissue [39]. Subcutaneous fat mass is associated with protective lipid and glucose profiles, as well as decreased cardiovascular and metabolic risks. Indeed, WHR may have difficulty in discriminating subcutaneous and visceral adipose tissue. The difference between male and female adolescents in our results may be explained by sexual dimorphism in body fat distribution or ethnic/race factors.

To the best of our knowledge, this is the first study to show that CONW have higher insulin resistance than NW particularly in Korean male adolescents. WHR can discover CONW not found by BMI. WHR is comparable to BMI in assessing MS components and is a good index for detecting insulin resistance even in normal weight male adolescents. Further researches to establish more appropriate cut-off value of WHR for health risks of obesity and the sexual dimorphism in body fat distribution on insulin resistance in adolescents are necessary. Screening for central obesity using WHR in clinical setting is recommended.

## Figures and Tables

**Table 1:** Subject characteristics and laboratory data according to four body composition groups in 13–18-year-old female adolescents.

Female	Normal weight (BMI 5th to 85th)		*P* value	Overweight (BMI over 85th)		*P* value	*P* value
	NW	CONW		OW	COOW		
	604 (72.7%)	83 (9.6%)	NW versus CONW	20 (2.5%)	125 (15.1%)	OW versus COOW	Among groups

Age (years)	15.4 ± 0.1	15.4 ± 0.2	0.879	15.6 ± 0.4	15.3 ± 0.2	0.579	0.945
Height (cm)	160.5 ± 0.3	158.7 ± 1	0.074	161.9 ± 0.8	160.8 ± 0.5	0.227	0.082
Weight (kg)	50.8 ± 0.2	54.9 ± 1.0	<0.0001	65.1 ± 1.2	68.6 ± 1	<0.022	<0.0001
BMI (kg/m^2^)	19.7 ± 0.1	21.7 ± 0.2	<0.0001	24.8 ± 0.3	26.5 ± 0.3	<0.0001	<0.0001
WC (cm)	65.8 ± 0.2	75.1 ± 0.6	<0.0001	71.3 ± 0.6	81 ± 0.7	<0.0001	<0.0001
WHR	41 ± 0.1	47.3 ± 0.2	<0.0001	44.0 ± 3.0	50.4 ± 0.4	<0.0001	<0.0001
SBP (mmHg)	101.8 ± 0.5	102.1 ± 1.4	0.813	102.2 ± 2.8	105.7 ± 1	0.242	<0.006
DBP (mmHg)	65.7 ± 0.4	64.7 ± 1.2	0.374	66.6 ± 2	66.7 ± 1	0.953	0.568
Glucose (mg/dL)	87.5 ± 0.3	88 ± 0.7	0.401	87.5 ± 1.2	89.9 ± 0.8	0.081	<0.028
Insulin (*μ*U/mL)	11.4 (11–11.8)	12.9 (11.9–13.9)	<0.006	14.3 (12.0–17.1)	16 (14.5–17.7)	0.272	<0.0001
HOMA-IR	2.5 (2.4–2.5)	2.8 (2.6–3)	<0.006	3.1 (2.6–3.7)	3.5 (3.2–3.9)	0.201	<0.0001
TG (mg/dL)	71.6 (68.7–74.7)	77.5 (69.5–86.6)	0.186	77.3 (59.2–100.9)	89 (80.0–99.1)	0.344	<0.002
HDL (mg/dL)	56.4 ± 0.5	54.6 ± 1.5	0.284	50.8 ± 2.1	49.4 ± 1.2	0.563	<0.0001
TG/HDL	1.3 (1.2–1.4)	1.5 (1.3–1.7)	0.121	1.5 (1.2–2.1)	1.8 (1.6–2.1)	0.285	<0.0001
ALT (U/L)	10 (9.7–10.3)	11.5 (10.8–12.3)	<0.001	11.8 (9.9–14.1)	12.9 (11.6–14.3)	0.392	<0.0001
AST (U/L)	16 (15.7–16.3)	15.6 (14.8–16.5)	0.458	15.6 (13.9–17.5)	15.8 (15.3–16.4)	0.839	0.861
WBC (10^9^/L)	5.9 (5.8–6)	6.1 (5.7–6.6)	0.260	6.3 (5.7–7.0)	6.7 (6.4–7.0)	0.351	<0.0001

Data are presented as the means ± standard error (SE), geometric mean (95% CI), or % (SE).

Insulin, HOMA-IR, TG, TG/HDL, ALT, AST, and WBC count were tested after logarithmic transformation.

NW, no central obesity normal weight; CONW, central obesity normal weight; OW, no central obesity overweight; COOW, central obesity overweight; WC, waist circumstance; WHR, waist to height ratio; BMI, body mass index; SBP, systolic blood pressure; DBP, diastolic blood pressure; HOMA-IR, homeostasis model assessment-insulin resistance; TG, triglyceride; HDL, high-density lipoprotein; ALT, alanine aminotransferase; AST, aspartate aminotransferase; WBC, white blood cell.

**Table 2:** Subject characteristics and laboratory data according to four body composition groups in 13–18-year-old male adolescents.

Male	Normal weight (BMI 5th to 85th)		*P* value	Overweight (BMI over 85th)		*P* value	*P* value
	NW	CONW		OW	COOW		
	662 (72.3%)	61 (7.0%)	NW versus CONW	21 (2.2%)	165 (18.5%)	OW versus COOW	Among groups

Age (years)	15.5 ± 0.1	15.3 ± 0.3	0.464	16.3 ± 0.4	15.5 ± 0.1	0.044	0.107
Height (cm)	171.6 ± 0.3	169.1 ± 1.4	0.099	176.9 ± 1.2	172.1 ± 0.6	<0.001	<0.0001
Weight (kg)	59.9 ± 0.4	68 ± 1.2	<0.0001	79.5 ± 1.2	81.8 ± 1	0.157	<0.0001
BMI (kg/m^2^)	20.3 ± 0.1	23.7 ± 0.1	<0.0001	25.4 ± 0.1	27.5 ± 0.2	<0.0001	<0.0001
WC (cm)	70.1 ± 0.3	82.2 ± 0.6	<0.0001	80.2 ± 1	88.6 ± 0.7	<0.0001	<0.0001
WHR	40.9 ± 0.1	48.7 ± 0.3	<0.0001	45.3 ± 0.4	51.4 ± 0.4	<0.0001	<0.0001
SBP (mmHg)	108 ± 0.5	110.4 ± 1.3	0.075	110.6 ± 2.4	115.8 ± 1.1	0.050	<0.0001
DBP (mmHg)	67.7 ± 0.4	65.7 ± 1.1	0.101	69.6 ± 1.6	70.7 ± 0.9	0.526	<0.002
Glucose (mg/dL)	88.6 ± 0.3	89.1 ± 1.1	0.642	91.2 ± 2.1	90.4 ± 0.6	0.702	<0.034
Insulin (*μ*U/mL)	10.6 (10.3–11)	13.6 (12.3–15.1)	<0.0001	15.1 (12.3–18.5)	17.4 (16.0–18.8)	0.210	<0.0001
HOMA-IR	2.3 (2.3–2.4)	3 (2.7–3.3)	<0.0001	3.4 (2.7–4.3)	3.9 (3.6–4.2)	0.288	<0.0001
TG (mg/dL)	70.4 (67.4–73.5)	76.2 (64.0–90.8)	0.386	73.6 (56.3–96.2)	114 (101.5–127.9)	<0.003	<0.0001
HDL (mg/dL)	51.8 ± 0.4	47 ± 1.1	<0.0001	52.6 ± 2.9	45.7 ± 0.7	<0.022	<0.0001
TG/HDL	1.4 (1.3–1.4)	1.6 (1.3–2.0)	0.101	1.4 (1.0–2.0)	2.5 (2.2–2.9)	<0.003	<0.0001
ALT (U/L)	12.8 (12.3–13.4)	17 (15.3–18.8)	<0.0001	22.8 (15.0–34.8)	24.8 (22.3–27.7)	0.704	<0.0001
AST (U/L)	17.9 (17.5–18.4)	18.6 (17.5–19.8)	0.272	20.1 (16.6–24.5)	21 (19.9–22.1)	0.698	<0.0001
WBC (10^9^/L)	6 (5.9–6.1)	6.4 (6.1–6.8)	<0.021	6.5 (6.1–6.9)	6.8 (6.5–7.0)	0.257	<0.0001

Data are presented as the means ± standard error (SE), geometric mean (95% CI), or % (SE).

Insulin, HOMA-IR, TG, TG/HDL, ALT, AST, and WBC count were tested after logarithmic transformation.

NW, no central obesity normal weight; CONW, central obesity normal weight; OW, no central obesity overweight; COOW, central obesity overweight; WC, waist circumstance; WHR, waist to height ratio; BMI, body mass index; SBP, systolic blood pressure; DBP, diastolic blood pressure; HOMA-IR, homeostasis model assessment-insulin resistance; TG, triglyceride; HDL, high density lipoprotein; ALT, alanine aminotransferase; AST, aspartate aminotransferase; WBC, white blood cell.

**Table 3:** Correlations between WHR or BMI and metabolic syndrome components in 13–18-year-old male and female adolescents.

	Female				Male			
	WHR		BMI		WHR		BMI	
	*r*	*P* value	*r*	*P* value	*r*	*P* value	*r*	*P* value

BMI	0.84	<0.0001			0.91	<0.0001		
SBP (mmHg)	0.18	<0.0001	0.18	<0.0001	0.27	<0.0001	0.33	<0.0001
DBP (mmHg)	−0.01	0.916	0.05	0.412	0.11	0.023	0.19	<0.0001
Glucose (mg/dL)	0.11	0.085	0.12	0.072	0.08	0.048	0.07	0.071
Insulin (*μ*U/mL)	0.30	<0.0001	0.33	<0.0001	0.49	<0.0001	0.48	<0.0001
HOMA-IR	0.30	<0.0001	0.32	<0.0001	0.47	<0.0001	0.46	<0.0001
TG (mg/dL)	0.17	<0.0001	0.16	<0.0001	0.32	<0.0001	0.31	<0.0001
HDL (mg/dL)	−0.22	<0.0001	−0.23	<0.0001	−0.27	<0.0001	−0.27	<0.0001
TG/HDL	0.22	<0.0001	0.22	<0.0001	0.35	<0.0001	0.34	<0.0001
ALT (U/L)	0.32	<0.0001	0.33	<0.0001	0.53	<0.0001	0.52	<0.0001
AST (U/L)	−0.004	0.919	−0.02	0.643	0.24	<0.0001	0.19	<0.0001
WBC (10^9^/L)	0.16	<0.0001	0.19	<0.0001	0.26	<0.0001	0.27	<0.0001

Data are presented as the means ± standard error (SE), geometric mean (95% CI), or % (SE).

Insulin, HOMA-IR, TG, TG/HDL, ALT, AST, and WBC count were tested after logarithmic transformation.

WHR, waist to height ratio; BMI, body mass index; SBP, systolic blood pressure; DBP, diastolic blood pressure; HOMA-IR, homeostasis model assessment-insulin resistance; TG, triglyceride; HDL, high density lipoprotein; ALT, alanine aminotransferase; AST, aspartate aminotransferase; WBC, white blood cell.

**Table 4:** Correlations between WHR and metabolic syndrome components of normal and overweight 13–18-year-old male and female adolescents.

	Female				Male			
	Normal weight		Overweight		Normal weight		Overweight	
	(BMI 5th to 85th)		(BMI over 85th)		(BMI 5th to 85th)		(BMI over 85th)	
	*r*	*P* value	*r*	*P* value	*r*	*P* value	*r*	*P* value

BMI	0.67	<0.0001	0.75	<0.0001	0.80	<0.0001	0.84	<0.0001
SBP (mmHg)	0.05	0.271	0.30	<0.001	0.10	<0.018	0.15	0.133
DBP (mmHg)	−0.08	0.063	0.06	0.516	−0.03	0.555	0.12	0.274
Glucose (mg/dL)	−0.02	0.557	0.21	0.160	0.01	0.772	−0.04	0.648
Insulin (*μ*U/mL)	0.10	<0.014	0.24	<0.006	0.27	<0.0001	0.26	<0.001
HOMA-IR	0.08	<0.025	0.26	<0.002	0.29	<0.0001	0.24	<0.003
TG (mg/dL)	0.07	0.059	0.12	0.145	0.10	0.064	0.23	<0.005
HDL (mg/dL)	−0.10	<0.017	−0.10	0.229	−0.17	<0.0001	−0.17	<0.036
TG/HDL	0.10	<0.015	0.13	0.073	0.140	<0.008	0.23	<0.005
ALT (U/L)	0.23	<0.0001	0.19	<0.034	0.32	<0.0001	0.33	<0.0001
AST (U/L)	−0.01	0.839	0.07	0.483	0.07	0.179	0.27	<0.0007
WBC (10^9^/L)	0.06	0.156	0.10	0.198	0.15	<0.001	0.17	<0.038

Data are presented as the means ± standard error (SE), geometric mean (95% CI), or % (SE).

Insulin, HOMA-IR, TG, TG/HDL, ALT, AST, and WBC count were tested after logarithmic transformation.

BMI, body mass index; SBP, systolic blood pressure; DBP, diastolic blood pressure; HOMA-IR, homeostasis model assessment-insulin resistance; TG, triglyceride; HDL, high density lipoprotein; ALT, alanine aminotransferase; AST, aspartate aminotransferase; WBC, white blood cell.

**Table 5:** Multivariate logistic regression analyses of having high insulin resistance among body composition groups.

	Model 1: adjusted for age			Model 2: adjusted for age and weight			Model 3: adjusted for age, weight, and ALT		
	OR	95% CI	*P* value	OR	95% CI	*P* value	OR	95% CI	*P* value
Female									

Group			<0.0001			0.118			0.151
NW	1			1			1		
CONW	1.89	1.04–3.42		1.54	0.85–2.78		1.51	0.83–2.75	
OW	3.09	1.22–7.85		1.56	0.55–4.41		1.55	0.54–4.44	
COOW	5.09	3.21–8.10		2.14	1.09–4.20		2.11	1.06–4.18	
Age	0.97	0.87–1.09	0.653	0.91	0.81–1.03	0.131	0.91	0.81–1.03	0.128
Weight				1.05	1.02–1.09	<0.003	1.05	1.02–1.09	<0.004
ALT							1.23	0.73–2.06	0.440

Male									

Group			<0.0001			<0.002			<0.022
NW	1			1			1		
CONW	4.37	2.32–8.22		2.82	1.38–5.75		2.46	1.21–4.99	
OW	8.29	2.43–28.22		3.29	0.90–11.98		2.57	0.78–8.49	
COOW	10.08	6.69–15.20		3.25	1.60–6.59		2.38	1.20–4.70	
Age	0.99	0.89–1.11	0.873	0.85	0.73–0.99	<0.045	0.82	0.70–0.95	<0.007
Weight				1.06	1.03–1.09	<0.0001	1.05	1.03–1.08	<0.0001
ALT							2.14	1.46–3.15	<0.0001

HOMA-IR, homeostasis model assessment-insulin resistance; CI, confidential interval; NW, no central obesity normal weight; CONW, central obesity normal weight; OW, no central obesity overweight; COOW, central obesity overweight; ALT, alanine aminotransferase.
